# New Insight on Phenolic Composition and Evaluation of the Vitamin C and Nutritional Value of Smoothies Sold on the Spanish Market

**DOI:** 10.3390/molecules27238229

**Published:** 2022-11-25

**Authors:** María del Carmen Razola-Díaz, Eduardo Jesús Guerra-Hernández, Belén García-Villanova, Vito Verardo

**Affiliations:** 1 Department of Nutrition and Food Science, Campus of Cartuja, University of Granada, 18011 Granada, Spain; 2 Institute of Nutrition and Food Technology “José Mataix”, Biomedical Research Center, University of Granada, Avda del Conocimiento sn., Armilla, 18100 Granada, Spain

**Keywords:** fruit smoothies, phenolic compound, antioxidant capacity, ascorbic acid, dehydroascorbic acid, phenolic acids

## Abstract

Fruits and vegetables are a source of a wide range of nutrients, including bioactive compounds. These compounds have great biological activity and have been linked to the prevention of chronic non-communicable diseases. Currently, the food industry is developing new products to introduce these compounds, whereby smoothies are becoming more popular among consumers. The aim of this study was to evaluate the nutritional quality and the polyphenol and vitamin C content of smoothies available on the Spanish market. An evaluation of the nutritional information and ingredients was carried out. The phenolic compounds were determined by HPLC-ESI-TOF-MS; the vitamin C content was quantified using HPLC-UV/VIS; and the antioxidant activity was analyzed by DPPH and FRAP. Among all of the ingredients of the smoothies, coconut and banana have shown a negative impact on the polyphenol content of the smoothies. In contrast, ingredients such as orange, mango, and passion fruit had a positive correlation with the vitamin C content. Moreover, apple and red fruits showed the highest positive correlations with most of the phenolic acids, flavonoids, total phenolic compounds, and antioxidant activities. In addition, a clustering analysis was performed, and four groups were clearly defined according to the bioactive composition determined here. This research is a precious step for the formulation of new smoothies and to increase their polyphenol quality.

## 1. Introduction

The Mediterranean diet encompasses the set of dietary patterns that occur in the different countries found in the Mediterranean Sea basin [1]. It is characterized by a high consumption of whole grain cereals, fruits, vegetables, nuts, legumes, olive oil, fish and a moderate consumption of meat and derivatives. Numerous epidemiological studies have observed that good adherence to this diet is related to a lower risk of cardiovascular disease, diabetes, and cancer, among others diseases [2,3,4]. However, this diet is being increasingly abandoned, and together with other unhealthy lifestyle factors, such as a decrease in physical activity and a more sedentary lifestyle, smoking or the consumption of ultra-processed food and very calorically dense foods, foods rich in sugars, refined flours, red meats, and a very limited consumption of fruits and vegetables [5], this is having repercussions on the health of the population and producing an increase in cases of chronic non-communicable diseases (NCD) [6,7,8,9,10]. NCDs can be defined as those pathologies of multifactorial origin and whose evolution is slow and lasts over time [11]. Among the pathologies considered we find cardiovascular disease, diabetes, chronic respiratory diseases, and some types of cancer.

Fruits and vegetables are low-calorie foods but with appreciable quantities of carbohydrates, fiber, minerals, water-soluble vitamins (vitamin B complex and vitamin C). and bioactive compounds such as flavonoids, carotenoids, tannins, etc [12]. Numerous studies have shown that a diet rich in fruits and vegetables decreases the risk of NCDs and increases life expectancy [13]. This effect is associated with the antioxidant capacity mainly attributable to bioactive compounds, including phenolic compounds and vitamin C [14,15].

Bioactive compounds derived from the secondary metabolism of plants, whose purpose is to act as a defense mechanism against pathogens or predators, function in plant reproduction, provide color, etc [16]. There are more than 5000 described compounds and they are present in vegetables, fruits, whole grain cereals, and other plant-based foods, although many remain unidentified [17]. They can be nitrogenous compounds, sulfur compounds, alkaloids, terpenoids, phenolic compounds, vitamins, and carotenoids, among others [17,18]. Fruit phenolic compounds have been related to the reduction of cardiovascular disease, diabetes mellitus, and mortality. In addition, they have been linked to better endothelial function and higher bone density [19]. The principal function of vitamin to highlight of is its role as a cofactor of numerous enzymatic processes. These enzymes catalyze a wide variety of hydroxylation reactions and are involved in the synthesis of collagen (essential to prevent scurvy) and carnitine, in the catabolism of thyroxine, and in the demethylation of proteins, DNA, and RNA [20,21]. Moreover, Vitamin C in its form of ascorbic or dehydroascorbic acid has been reported to reduce cellular oxidative stress with all the positive effects that it can have in the human health [21,22,23]. The main sources of vitamin C are fruits and vegetables, preferably fresh. This is due to the fact that vitamin C is characterized as being water-soluble and very thermolabile, so the processing of these foods implies great losses. Among the foods that have higher amounts of vitamin C, mangos, kiwis, cauliflowers, red peppers, Brussels sprouts, or grapefruit stand out, among others [24].

The consumption of fruits and vegetables is the main basis of a healthy and varied diet. The World Health Organization (WHO) recommends that the daily consumption of this type of food should be greater than 400 g/day or at least five pieces of fruit and vegetables per day [25]. Nowadays, the daily intake in the general population is much lower than recommended. Additionally, in some studies it has been observed that during the COVID-19 pandemic, the consumption of canned foods, whose shelf life is longer, increased while the consumption of fresh fruits and vegetables decreased [26]. This is linked to the fact of that the accessibility of these fresh products is more limited, since they are more expensive and highly perishable foods that need adequate storage. That is why the food industry, together with a greater demand for healthy and good quality products, is developing new methods of consuming fruits and vegetables, alternative to the traditional ones, as a way to increase and/or maintain the recommended consumption of these foods.

In this context, some products that are becoming more and more popular are smoothies. These drinks were first introduced in the United States in the 1960s and later, in the 2000s, they became popular again [27]. Smoothies are defined as non-alcoholic beverages prepared from fruits and/or vegetables, fresh or frozen, which are crushed until they achieve a homogeneous appearance [28,29]. In fact, the name “smoothie” comes from the English “smooth” due to its appearance, since this type of drink is not filtered, unlike juices, so that it preserves the pulp and fiber, giving it a denser, more even appearance [28]. In addition, sometimes other types of ingredients are added to these drinks apart from fruit and vegetables, such as cereals, ice, or dairy products (milk, yogurt, etc.).

In the process of making juices and smoothies, the cellular structures of the fruits and vegetables break, leading to the release of enzymes that, together with the microorganisms that are present, make the product more susceptible to degradation, compromising its useful life [30,31]. To increase the shelf life, the smoothie industry has used very intense heat treatments in order to ensure the inactivation of enzymes and the reduction of the microbial load of foods. In addition, other compounds such as vitamins and phenolic compounds are also released from the cell matrix. These molecules are of great nutritional interest due to their health benefits, however, some can degrade, thus reducing their nutritional and organoleptic quality [28]. Therefore, the main challenge for the industry in the production of smoothies is the implementation of adequate conservation and sanitization methods, so that the shelf life of the product increases, without affecting the organoleptic and nutritional characteristics [32].

Thus, the main objective of this work was to evaluate the nutritional quality of the smoothies currently available on the Spanish market in terms of phenolic compounds, vitamin C content, and antioxidant activity, as a precious step to formulate new products and increase their polyphenol quality.

## 2. Results and Discussion

### 2.1. Nutritional Evaluation of the Smoothies

**Figure 1:**
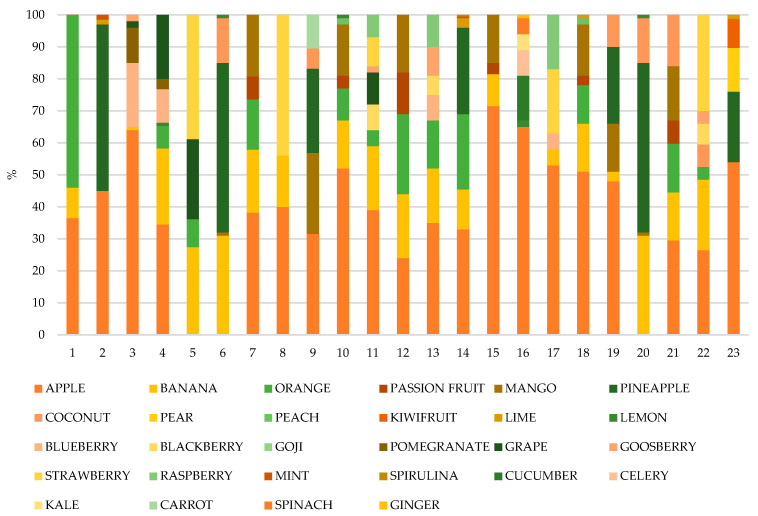
Ingredients of the evaluated smoothies expressed as percentage.

The smoothies evaluated were characterized by being drinks composed mainly of juice and purées of different fruits and vegetables. In some cases, other compounds such as chlorophylls, concentrates, or spirulina extract were also added to these smoothies. The ingredient composition of the smoothies is represented in Figure 1 expressed as percentage according to the information given in their labelling.

Most of the products analyzed contained apple-based ingredients in their composition, either in the form of juice and/or purée. More specifically, of the 23 smoothies analyzed, 20 included apples among their ingredients, and in 19 of them, it was among the major two ingredients. Other ingredients also widely used in the preparation of smoothies were bananas, oranges, and strawberries. The marketed volume of this product is generally 250 mL and on certain occasions it can reach 330 mL. Table 1 collected in a nutshell the nutritional composition of the studied smoothies in terms of energy and macronutrients as reported in their labelling for 100 mL of product, the measured pH, and °Brix.

In relation to the nutritional value of smoothies, globally, we can see that they are drinks whose energy intake ranges between 38 kcal and 82 kcal per 100 mL of product (Table 1). The smoothie that had the lowest caloric intake was number 16 and the highest caloric intake corresponds to number 7. A significant (*p* < 0.05) positive correlation was found between energy content and banana content, an ingredient that was present in an amount ≥ 20% in all smoothies that had ≥ 71 kJ (4, 7 and 20). Likewise, a significant (*p* < 0.05) negative correlation was found with the apple content, despite the fact that this is a major ingredient in many of the smoothies, thus corroborating that it is a low-calorie fruit that does not impact the energy supply in the smoothies.

With regards to macronutrients, it should be noted that smoothies are drinks that stand out mainly for their carbohydrate content. The contribution of total carbohydrates varies according to the product, but the sampled smoothies ranged between 7.9 and 14.6 g per 100 mL (Table 1). The major carbohydrate forms in the smoothies were sugars reaching in some cases 100% of the present carbohydrates. Moreover, the rest of the carbohydrates that are not free sugars in some smoothies could be mainly complex carbohydrates (oligosaccharides and polysaccharides). It should also be noted that none of the smoothies analyzed had added sugar, so all the sugars that appeared on the nutritional labels were naturally present. Non-caloric sweeteners were also not added to any of the products. This may be due to the fact that smoothies, in general, are products made up mostly of fruits—foods that are characterized by their sugar content and sweet taste. The ingredients that showed the greatest significant positive correlation (*p* < 0.1) in the intake of carbohydrates were oranges (r = 0.4401), mangos (r = 0.4588), and bananas (r = 0.3907), as well as in the content of total sugars. In fact, the smoothie with the highest carbohydrate content was number 12 and it contained 25% orange as the main ingredient, as well as 18% mango and 20% banana. Meanwhile, the smoothie with the lowest content was number 16, which was characterized by not including any of these three ingredients in its composition.

Moreover, the studied smoothies were not characterized by a high fiber content. The oscillation range of the fiber content was found between 0.3 and 1.8 g/100 mL of product. The ingredients that contributed significantly (*p* < 0.1) to fiber intake were apples (r = 0.4525) and raspberries (r = 0.3853). In fact, all the smoothies that reported fiber > 1 g/100 mL had those ingredients. It is important to point out that not all of the smoothies had the fiber information present, since it is not mandatory for it to be reflected in the nutritional labelling according to regulation (EU) N° 1169/2011 of the European Parliament and of the Council of 25 October 2011 on the provision of food information to consumers.

On the other hand, the amount of protein and fat was generally insignificant, since the raw materials used in the preparation of smoothies are not characterized as foods rich in protein or lipids. Even so, it is noteworthy that 4 of the 23 products analyzed contained a greater amount of fat, reaching 3.2 g per 100 mL of product. This can be associated with the fact that these smoothies, among their ingredients, contained coconut in different variants, such as coconut milk, a coconut drink, or coconut-based preparation which contains coconut pulp and water. It could be explained by the fact that coconuts are a fruit rich in fats, mainly medium and long chain saturated fats, which increase the lipid content and, consequently, also the energy value of the products to which it is added [33]. This was also confirmed statistically as coconuts were shown to have a significantly strong correlation (*p* < 0.05) with total fat (r = 0.7167) and saturated fat (r = 0.7430) content.

In addition to the nutritional labeling, the pH and °Brix were measured experimentally. As expected, the smoothies analyzed presented an acidic pH. The product with the lowest pH was number 17 at 3.31 and the smoothie with the highest pH was number 20 with a pH of 4.06. The results found were in concordance with other studies that previously reported a pH range between 3 and 4 [31,34,35,36,37,38]. This acidic pH is very useful when it comes to ensuring microbiological stability, which together with pasteurization treatments or high-pressure processing prevents the proliferation of pathogenic microorganisms and increases the shelf life of the product [37]. Ingredients such as apples, oranges, lemons, passion fruit, mangos, kiwifruit, mint and red fruits showed a negative correlation with the pH which means that they are good ingredients to add to the smoothies to help to avoid the microorganisms’ spoilage by reducing the pH.

In relation to the number of soluble solids, it can be seen that the range of variation went from 9 °Brix, which corresponds to smoothie number 17, to 15 °Brix which corresponds to the smoothies that mostly contained pineapple, coconut and banana (6 and 20). The obtained results are in agreement with previous studies [27,31,37,38,39]. It is interesting to mention that the smoothies with a higher pH and soluble solids content also had a greater amount of coconut or coconut-based preparations in their composition, with the amount being up to 14%. This has also been seen statistically with a significant (*p* < 0.05) positive correlation (r = 0.7953) between the total soluble solids and the coconut content.

### 2.2. Identification of Polar Compounds by HPLC-ESI-TOF-MS

A total of 40 polar compounds have been tentatively identified in the smoothies. Between them, four are organic acids, three are hydroxybenzoic acids, five hydroxycinnamic are acids, twenty-one are flavonoids, and seven of them are other metabolites. Table 2 shows an overview of all of the proposed compounds with their retention time (min), molecular formula, experimental and calculated *m/z*, and *m/z* in source fragments. Furthermore, all of the metabolites showed a score higher than 90% and an error (ppm) lower than 5. These parameters were given by the software MassLynx 4.1. To identify the compounds, the generated molecular formulas and some in-source fragments were checked, studied, and compared with different databases such as PubChem, Mass bank, Phenol-Explorer, and the literature. Some representative total ion chromatographs of the analyzed smoothies are shown in Figure S1 from Supplementary Materials.

Four organic acids were identified corresponding to n° 1, 2, 5 and 40, and they were named as malic acid, citric acid, isopropylmalic acid, and pinellic acid. Malic acid and its derivatives are the main acids present in many fruits, including apricots, blackberries, blueberries, cherries, grapes, peaches, pears, plums, and quince; similarly citric acid is extensively present in citric fruits such as oranges and lemons, among others. Both acids are two types of compounds that are also natural, safe additives, which are widely used in food, medicine, daily chemical product, and health product industries [40]. 3-isopropylmalic acid was identified in concordance with Ricciutelli et al. [41] who described it with the *m/z* 175 and the main *m/z* fragment 113 that corresponds to the loss of CO_2_ and H_2_O [M-CO_2_-H_2_O]^−^. Otherwise, pinellic acid is a metabolite of linoleic acid, one of the major fatty acids found in lipids. It has a role as an adjuvant and an anti-inflammatory agent and it is being increasingly found in fruits and vegetables [42].

A total of eight phenolic acids were found in the analyzed smoothies. At 0.753 min, gallic acid previously reported in grapefruit, bananas, and pomegranates, among others, was found [43]. With the molecular formula C_7_H_6_O_4_, two isomers of protocatechuic acid were found corresponding to n° 4 and 7, previously reported in the highest amount in apples [44] and dates [45]. Three isomers of chlorogenic acid were detected with the *m/z* 353 (n° 9, 10 and 12) reported previously in berries, apples, bananas, citrus fruits, and pears, among others [46]. Moreover, at 4.952 and 5.155 min, two isomers of coumaroylquinic acid were tentatively identified. Both have been extensively reported in fruits and vegetables [43].

The major group of identified compounds are flavonoids. The flavan-3-ols catechin and epicatechin were identified with the *m/z* 289 at 3.32 and 4.69 min, respectively. Quercetin derivatives have been found corresponding to compound numbers 17, 20, 22, 26, and 28 named as quercetin dihydrate, isoquercetin, hyperoside, quercetin 3-O-beta-D-xylopyranoside and quercetin 3-rhamnoside, respectively. Those compounds have been found in fruit matrices several times [43,47]. Moreover, phloretin 2′-xyloglucoside and phloridzin, two compounds characteristic of apples were identified at 8.363 and 9.137 min [48]. Additionally, the compounds named as narirutin, naringin, hesperidin, didymin, narigenin, and hesperetin (n° 16, 19, 25, 31, 35 and 37, respectively) were identified, which were extensively reported to be found in citrus fruits [49]. Corresponding with n° 8 and 11, two isomers of a kaempferol derivative were identified and named as kaempferol 3-[2′′′,3′′′,5′′′-triacetyl-alpha-L-arabinofuranosyl-(1→6)-glucoside isomers a and b. With the molecular formulas C_21_H_20_O_11_ and C_21_H_18_O_11_, the compounds kaempferol-3-glucoside and kaempferol-3-glucuronide were found, respectively. Other kaempferol derivatives were found with the *m/z* 489 and the *m/z* fragments 285, 255, and 227 at 10.942 and 11.511 min which were named as kaempferol 3-(6-acetylgalactoside) isomers a and b, respectively. All of those kaempferol derivatives have been previously described and found in fruits [50,51,52].

In addition, other metabolites were identified in the smoothies. Two isomers of syringin, a phenylpropanoid glycoside, were detected with the *m/z* 371 (n° 18 and 23), and a derivative named as methylsiringin at 13.24 min, previously found in caraway, fennels, and lemons [43]. In relation to the terpenoids, four limonoids have been identified in accordance with Gualdani et al. [53] and Shi et al. [54], who characterized limonoids from several citrus species and matrixes. Those compounds are named nomilin glucoside, nomilinic acid 17-beta-D-glucopyranoside, limocitrin, and 7-acetoxy-6-hydroxylimonin corresponding to n° 32, 34, 35, and 39, respectively.

### 2.3. Quatification of Phenolic Compounds by HPLC-ESI-TOF-MS

The phenolic acids and flavonoids identified in the smoothies were quantified, and the results are summarized in Table 3. As can be seen, the sum of phenolic acids ranged between 18.0–5561.5 µg/mL and the total flavonoids ranged between 67.8–4200.2 µg/mL with variation coefficients of 68.7 and 59.2%, respectively. The variation between the samples is very big depending on the smoothie.

The major phenolic acid in most of the smoothies was the total chlorogenic acid, a fact attributable to the presence of apple in all of the smoothies showing a significant (*p* < 0.05) positive correlation of 0.8185. In smoothies 5, 6, and 20, the only phenolic acid quantifiable was gallic acid. The strongest significant positive correlation with gallic acid was found with passion fruit (r = 0.6387), mango, (r = 0.6993) and peach (r = 0.4571) and smoothie 10 was the only one that contained those three ingredients accounting for 22% of the content of the smoothie which had the highest amount of this hydroxybenzoic acid. The smoothie that had the highest content of total protocatechuic acid was number 4 which had grape and blueberry in 30% of the smoothie, ingredients that showed a significant (*p* < 0.05) positive correlation with this compound. For the total coumaroylquinic acid, smoothies 15 and 17 had the highest content and this was attributable to the apple and raspberry content that had a significant (*p* < 0.1) positive correlation with the compound. Regarding the flavonoids phloridzin and phloretin 2′-xyloglucoside showed a significative (*p* < 0.05) strong positive correlation (r = 0.7729 and 0.6467, respectively) with the content of apple in the smoothies. Moreover, some quercetin derivatives such as isoquercetin, quercetin 3-rhamnoside, and quercetin 3-O-beta-D-xylopyranoside showed a significative (*p* < 0.05) positive correlation with blueberry and pomegranate. Other compounds such as naringin, narirutin, hesperidin, didymin, hesperetin, and quercetin dihydrate showed a significative (*p* < 0.05) positive correlation with citrus fruits such as oranges, passion fruit and mangos, among others. In contrast, naringenin, catechin, and hyperoside had a significative (*p* < 0.05) positive correlation with red fruits such as blackberries, strawberries, grapes, currants and raspberries.

The smoothie that had the highest total phenolic compounds content was number 15 followed by 17, 3, and 23. Although they had different compositions, all of them had apple as the main ingredient in ≥ 53% of the smoothie. The fact that apple had a huge effect in the phenolic composition of the smoothies was also confirmed statistically, as shown by the significative (*p* < 0.05) positive strong correlation with the total phenolic acids (r = 0.8337), total flavonoids (r = 0.7964), and in the total phenolic content (r = 0.8398). Other ingredients that had a significative (*p* < 0.05) positive influence on the flavonoid composition were blueberry (r = 0.5403) and pomegranate (r = 0.4803). In contrast, the smoothies that had the lowest polyphenol content were 20 < 6 < 5 < 9 < 22 which had in their ingredients: coconut, banana, pineapple, or a mix of them in a proportion of at least 27%. These three ingredients showed a significative (*p* < 0.1), moderately strong negative correlation with the flavonoid and phenolic acid content in the smoothies. As expected, those ingredients that are the main contributors to the fat and saturated fat content of the smoothies had a significative negative impact on the total phenol content, so it could be affirmed that the fats in the smoothies have a significative (*p* < 0.05) negative effect regarding the total polyphenol content (r = −0.4858).

Additionally, a significant (*p* < 0.05) negative correlation between the pH and the phenolic acids (r = −0.6127), flavonoids (r = −0.7268), and total phenolic compounds (r = −0.6816) was found. This explains that having an acidic pH of the smoothies is essential to avoid the degradation of the phenolic compounds in addition to reducing the microorganism spoilage.

Compared to other the authors, the relationship between a higher content of total polyphenols in those smoothies with a base or a greater amount of red fruits in their composition has been seen in other studies [38,55,56,57].

### 2.4. Vitamin C Content of Smoothies by HPLC-UV/VIS

**Figure 2:**
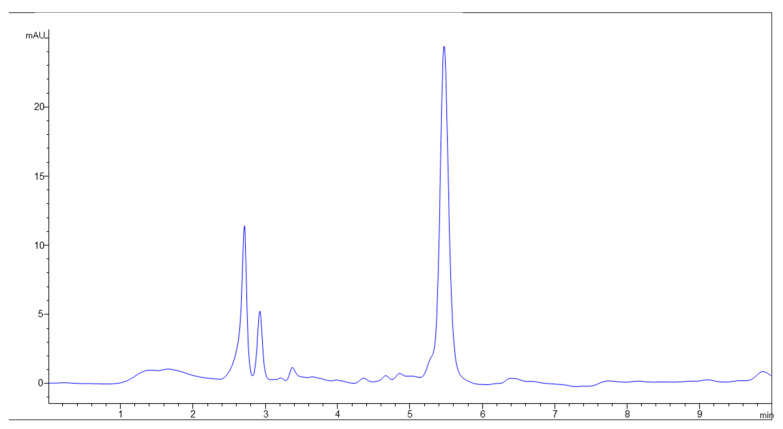
HPLC-UV/VIS chromatogram of vitamin C determined in smoothies.

Vitamin C, as we mentioned earlier, is a water-soluble vitamin that can be found in two forms in fruits and vegetables: AA (reduced form) and DHA (oxidized form). The oxidation of AA to DHA is a reversible reaction, so it is important to determine the amount of each one, since both forms are active in our body [58]. The AA and DHAA content were determined in the smoothies by HPLC-UV/VIS and the results obtained are presented in Table 4. Additionally, Figure 2 shows the typical chromatogram of the determination of vitamin C (peak at 5.5 min) by HPLC-UV/VIS in the smoothies.

As can be seen, the total vitamin C results ranged between 491.78 and 2660.02 µg AA/ mL smoothie. These results were similar to those obtained by Müller et al. (2010), whose vitamin C values in commercial smoothies ranged 42–95 mg AA/100 mL smoothie [55]. Moreover, Hurtado et al., (2015) [59] reported values of ≈ 40 mg AA/100 mL of total vitamin C in smoothies composed of apples, oranges, strawberries and bananas.

The smoothie that had the highest content of vitamin C was 9. It was the only one that presented carrots in its ingredients, and a significative (*p* < 0.05) strong correlation with the content of AA (r = 0.8439) and total vitamin C (r = 0.9222) was found. After it, smoothies 18 and 10 also presented the highest values of vitamin C. The three smoothies had the presence of mango in their composition in common. In fact, a moderately strong positive correlation (*p* < 0.05) was found between mango content and AA (r = 0.5658) and total vitamin C (r = 0.5447) content. In general, tropical fruits showed a positive correlation with ascorbic acid. Regarding the proportion of the AA and DHAA, it can be observed that AA was the majoritarian with a relation higher than 2:1 (AA: DHAA), except in some cases. There were some smoothies where all of the vitamin C content was in the form of AA, such as smoothie 1, 5, 6, and 18. Smoothie 4 had a very similar amount of AA and DHAA (relation 1.15) that could be due to the balance in its composition between apples and red fruits. In contrast, in smoothie 16, it was observed that the entire amount of total vitamin C was in the form of DHAA. This can be explained by the presence of a high amount of apple in combination with other minor ingredients such as cucumber, celery, kale and ginger, and no other fruits. Apple was the ingredient that showed a higher significant correlation (*p* < 0.1) with the total dehydroascorbic acid content.

### 2.5. Antioxidant Activity of Smoothies by DPPH and FRAP Assays

The antioxidant activity of the smoothies has been determined by two methods: DPPH and FRAP, and the results are shown in Table 5. Both methods showed a significant (*p* < 0.05) positive strong correlation among them (r = 0.8908).

With the DPPH method, the antioxidant capacity of the smoothies ranged from 220.5 to 1926.50 µg TE/ ml smoothie. Regarding the FRAP method, the capacity ranged between 1126.84 and 8167.37 µg TE/ ml smoothie. Overall the FRAP numerical results are higher than those obtained for the DPPH as observed by other authors [31,60]. This is mainly explained by the fact that the FRAP technique has more specific affinity for vitamin C than DPPH. It has also been appreciated statistically as the total vitamin C content exhibited a higher significant (*p* < 0.1) positive correlation with FRAP (r = 0.8004) than with DDPH (r = 0.3205). Similarly, Gonzalez-Tejedor et al. (2017) [61] observed that the method that obtained a better correlation with the concentration of antioxidant compounds such as ascorbic acid in smoothies was FRAP, followed by ABTS, and finally DPPH. Moreover, in our case, the content of total phenolic compounds showed a strong significant correlation (*p* < 0.05) with the values of 0.9359 and 0.8935 with the antioxidant activity measured with DPPH and FRAP, respectively. Going further, specific compounds such as catechin, hyperoside, and isoquercitrin showed significative (*p* < 0.05) positive correlations with the antioxidant activity, with it being stronger for DPPH (r = 0.5497, 0.6052, and 0.5624, respectively) than for FRAP (r = 0.5097, 0.4379, and 0.3777, respectively).

The smoothie that contained the least antioxidant activity was 16 by both methods. This smoothie is the one that is most different from the others because after having apple as the major ingredient, it contains cucumber, celery, kale and spinach, ingredients which showed a significant (*p* > 0.05) negative correlation with the antioxidant activity. On the opposite side, the ingredients that showed a clear significant and positive correlation with the antioxidant activity for both methods were blueberries, blackberries, pomegranates, currants, strawberries, raspberries and apples. This make sense as the smoothies that had the higher antioxidant activity were 3, 13, 11, 4, and 22 which contained red fruits in their ingredients. This trend was also observed in the analyses performed by Müller et al. (2010) [55] and Nowicka et al. (2017) [39] whereby smoothies with a greater amount of red fruits obtained a higher antioxidant capacity, while those composed of mango, banana, apple, and pear, among others, presented a lower capacity. This incremented antioxidant activity in these smoothies can be explained by the presence of tannins and anthocyanins present in those red fruits. Moreover, smoothie number 9 exhibited the highest antioxidant activity by the FRAP assay. This smoothie did not have red fruits in its ingredients but rather its ingredients were: apple purée, pineapple juice, mango purée, carrot juice, and coconut milk. The fact that this smoothie stands out from the rest in terms of antioxidant capacity may be due to the fact that it had the highest content of vitamin C. In both cases, tannins and vitamin C, as has been observed in previous studies, mainly influence the antioxidant capacity of smoothies [62]. Moreover, other factors can have an influence such as synergistic or antagonistic interactions between the phenolic compounds, increasing or decreasing the antioxidant capacity [38]. Other factors that can influence the antioxidant capacity of the product are processing or pasteurization treatments [63,64,65]. According to Škegro et al., (2021) [66] high-pressure processing exhibits greater stability of bioactive compounds than pasteurization during the shelf life of smoothies. Among all of the smoothies, only numbers 15, 16, and 17 were shown in the labelling to have been submitted to high-pressure processing, and smoothies 1–3, 12–14, and 18 were shown to have been pasteurized, but no correlations were found between this information and the antioxidant results.

### 2.6. Clustering Analysis

**Figure 3:**
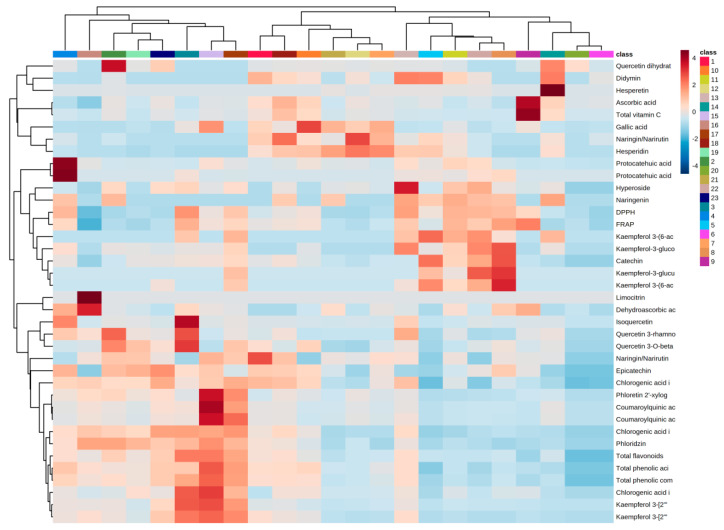
Clustering heatmap of the smoothies for the measured features.

A hierarchical clustering heatmap was performed to provide an intuitive visualization of all of the obtained data for the smoothies. The data used were previously normalized, the distance measure was the Pearson statistical measure, and the clustering method was the average. Therefore, the clustering result for the features and samples is shown in Figure 3. Each colored cell on the map corresponds to a concentration value normalized from 1 (intense red) to −1 (intense blue), with samples in columns and the features (phenolic compounds, vitamin C, and antioxidant activities) in rows. Moreover, each sample has an associated color from 1 to 23.

As can be seen from the figure, the samples can be classified into four groups according to the analysis performed here. The groups have been classified as group 1, the group formed by smoothies 6, 9, 14, and 20; group 2, formed by smoothies 5, 8, 11, 13, and 22; group 3, formed by smoothies 1, 7, 10, 12, and 18; and group 4, formed by the rest of the smoothies. Briefly, group 1 seems to be composed of the smoothies that had pineapple, coconut and banana in their ingredients, with those types of fruit being those that had the lower polyphenol content and the higher saturated fat content. Group 2 was formed by most of the smoothies that presented red fruits in their ingredients which had the higher antioxidant activity. In group 3, there are the smoothies whose major ingredients were tropical yellow fruits such as mangos, passion fruit and oranges, among others. Finally, group 4 was composed of all of the smoothies that had apple in bigger concentrations, with its characteristic phenolic compounds as such phloridzin in higher concentrations.

## 3. Materials and Methods

### 3.1. Chemicals and Samples

HPLC (high-performance liquid chromatography)-grade water and other reagents and solvents were purchased from Merck KGaA (Darmstadt, Germany).

Twenty-three smoothies were purchased in the main supermarkets in the urban area of Granada. All of the smoothies were obtained with similar dates of preferential consumption. The samples were kept at 4 °C, manually mixed to homogenize the content, and directly analyzed. Table 6 lists the product names of the 23 smoothies evaluated, including the numerical code assigned to each of them.

### 3.2. Determination pH and Soluble Solids

For the evaluation of pH, an automatic pH meter (Benchtop pH/ORP/ION Meters LAQUA pH1100, Horiba Scientific, Japan) was used and soluble solids were determined using a handheld refractometer (HR-130 hand refractometer 0–32% Brix ATC, Optika Microscopes, BG, Italy) and represented as degrees Brix (°Brix).

### 3.3. Determination of Polar Compounds by HPLC-ESI-TOF-MS

For the extraction of phenolic compounds, 8 mL of the homogenized smoothie was taken and lyophilized using a Zirbus lyophilizer (Bad Grund, Germany) for 120 h at −50 °C with a pressure of 0.4 mbar and reconstituted in 1 mL of methanol: water 1:1 (*v*/*v*). The extracts were filtered with regenerated cellulose filters 0.2 µm (Millipore, Bedford, MA, USA) and kept at −18 °C until the analyses.

The phenolic profile characterization and quantification of the smoothies was performed according to a previously described method [67]. The analyses were carried out in duplicate on an ACQUITY Ultra Performance LC system (Waters Corporation, Milford, MA, USA) coupled to an electro-spray ionization (ESI) source operating in the negative mode and a time-of-flight (TOF) mass detector (Waters Corporation, Milford, MA, USA). The compounds of interest were separated on an ACQUITY UPLC BEH Shield RP18 column (1.7 µm, 2.1 mm × 100 mm; Waters Corporation, Milford, MA, USA) at 40 ◦C using a gradient previously stated by Verni et al. [67] using water containing 1% acetic acid as mobile phase A and acetonitrile as mobile phase B. The gradient was: from 0 to 2.3 min, 1% B; 4.4 min, 7% B; 8.1 min, 14% B; 12.2 min, 24% B; 16 min, 40% B; 18.3 min, 100% B, 21 min, 100% B; 22.4 min, 1% B; 25 min, 1% B. The flow rate was established to 0.6 mL/min. The volume injection was 2 µL. Finally, external calibration curves were prepared for the quantification of phenolic compounds: vanillic acid, chlorogenic acid, ferulic acid, rutin, quercetin, catechin, phloretin, and phloridzin (Table S1 from Supplementary Materials). The analyses were performed in triplicate and the results are expressed as µg/ mL smoothie. The calibration curves were prepared from the limit of quantification (LOQ) to 250 µg/L. All calibration curves revealed a good linearity among different concentrations, and the determination coefficients of the linear regression were higher than 0.9 in all cases. The method used for the analysis showed a limit of detection (LOD) within the range of 2.94–7.57 µg/mL and the LOQ was within the range of 9.79–25.256 µg/mL. To assess the repeatability of the method, sample S6 has been injected five times in a day and for three consecutive days. The intraday repeatability (expressed as % RSDs) on the retention times ranged from 0.37 to 2.85%, whereas the interday repeatability was from 1.29 to 2.38%. The intraday repeatability (expressed as % RSDs) on the total peak area was 0.67%, whereas the interday repeatability was 1.99%.

### 3.4. Determination of Vitamin C Content by HPLC-UV/VIS

The ascorbic acid (AA) extraction was carried out according to the procedure reported by Mesías-García et al., (2010) [68]. Briefly, 0.5 mL of homogenized smoothie was mixed with 2.5 mL of 10% (*w*/*v*) metaphosphoric acid solution and then diluted with demineralized water to a final volume of 25 mL in a glass volumetric flask. The mixture was homogenized and centrifuged at 9000 rpm for 15 min (room temperature) (Centrifuge Universal 32, Hettich, Tuttlingen, Germany). The supernatant was filtered through 0.20 μm Millex filters (Millipore, Bedford, MA, USA), and the samples were then ready to be injected into the HPLC system.

In order to determine the total vitamin C content, the reduction of the dehydroascorbic acid (DHAA) to AA had to be performed. Then, 1 mL of the filtered sample from the AA analysis was added of 0.2 mL of a reductant agent, dithiothreitol (DTT) (1 mg/mL diluted in 45% K_2_HPO_4_). The mixtures were kept in darkness for 30 min at room temperature, and then the reduction was stopped by the addition of 0.2 mL of H_3_PO_4_ 2M and the samples were injected into the HPLC system.

The DHAA content was calculated by the difference between the vitamin C content (after DHAA reduction) and the initial AA content (prior to reduction). Both determinations (AA and vitamin C) were performed in triplicate and the results were expressed as µg AA/mL smoothie.

The HPLC system used in this study was equipped with a Varian Prostar model 325 ultraviolet detector. Samples were introduced into the column through an automatic injector equipped with a sample loop (20 μL). Separations were performed on a Gemini 5 μm C18 (150 × 4.6 mm) Phenomenex column for all of the compounds. The measurement was performed under isocratic conditions, using demineralized water acidified with sulfuric acid to pH 2.2 as the mobile phase at a flow rate of 0.6 mL/min, with a wavelength of 245 nm [68].

The standard curve of AA was elaborated (2.5, 5, 10, 25, 35, 50, 75, 85 and 100 µg/mL) and the equation obtained was y = 67.26x − 153.26 (R^2^ = 0.9979).

### 3.5. Antioxidant Assays: DPPH and FRAP

The antioxidant capacity of the smoothies was evaluated by two different methods. The DPPH assay was carried out by a method proposed by several authors [69,70]. In brief, 2.9 mL of DPPH was added to 100 µL of each sample and diluted 10 times, and after rapid stirring, the bleaching power of the extract was observed in a time interval from 0 to 30 min at 517 nm. FRAP scavenging activity was performed as described by Pulido et al., (2000) [71]. It is based on the reduction of Fe^3+^ to Fe^2+^ by the antioxidant substances. A total of 30 µL of each sample diluted 10 times was added to 90 µL of distilled water and 900 µL of the FRAP reagent. It was kept for 30 min at 37 ◦C and measured in the spectrophotometer at 595 nm. Standard curves of Trolox equivalents (TE) (1, 5, 10, 20, 50, 80, 100, 150, 200 µg/mL) were elaborated for each assay and the equations obtained were y = 0.002x + 0.0384 (R² = 0.9965) and y = 0.0019x + 0.1338 (R² = 0.9967) for the DPPH and FRAP assays, respectively. The analyses were performed in triplicate and the results are expressed as µg TE/ mL smoothie.

### 3.6. Data Processing

The data for the identification of polar compounds and the identification of phenolic compounds HPLC-ESI-TOF-MS were elaborated using MassLynx 4.1 software (Waters Corporation, Milford, MA, USA). Pearson’s correlation analysis between the smoothies’ ingredients, nutritional features, and antioxidant and phenol content (Figure S2 from Supplementary Materials) and the hierarchical clustering analysis (Figure 3) were performed using MetaboAnalyst 5.0.

## 4. Conclusions

In this work, the nutritional value, content of phenolic compounds, vitamin C content, and the antioxidant activity of different smoothies was studied. Analysis by HPLC-ESI-TOF-MS permitted the identification of 40 phenolic compounds. Among all of the ingredients of the smoothies, coconut and banana were those that showed the highest negative correlation with the total phenolic compounds and contributed the most to the energy, fat, saturated fat, and energy content of the smoothies. Ingredients such as oranges, mangos, and passion fruit had a positive correlation with gallic acid, some flavonoids, AA, and total vitamin C content despite being the ingredients that had the higher sugar and carbohydrate content. Apples and red fruits contributed the most to the fiber content with high positive correlations with most of the phenolic acids, flavonoids, and total phenolic compounds. Moreover, the smoothies that had the highest antioxidant activities were those that had apples, red fruits, or citrus fruits in their ingredients. All of the analyses performed suggest the relationship between the type of fruit and the content of phenolic compounds, vitamin C, and the antioxidant activity as shown in the clustering. Therefore, these data will be useful for selecting raw materials and/or for evaluating the impact of technology on the smoothies’ quality.

## Figures and Tables

**Table 1:** Summary of the nutritional labelling and physical–chemical analysis of the studied smoothies per 100 mL of product.

	Energy (kcal)	Energy (kJ)	Fats (g)	Saturated Fats (g)	Carbohydrates (g)	Sugars (g)	Fibre * (g)	Protein (g)	pH	°Brix
Average	56.3	237.5	0.5	0.4	11.8	10.9	0.7	0.6	3.61	12.53
Median	53.0	225.0	0.1	0.0	12.0	11.0	0.8	0.6	3.57	12.60
Min.	38.0	160.0	0.0	0.0	7.9	6.8	0.3	0.0	3.31	8.00
Max.	82.0	343.0	3.2	2.9	14.6	13.4	1.8	0.9	4.06	15.00
CV (%)	19.7	19.2	185.6	236.4	11.5	13.8	85.5	31.8	5.81	13.49

* Data of fiber content were not available for all of the samples.

**Table 2:** Identified compounds in the analyzed smoothies by HPLC-ESI-TOF-MS.

N°	Time (min)	*m/z* Experimental	*m/z* Calculated	Error (ppm)	Score (%)	Molecular Formula	*m/z* in Source Fragments	Compound
1	0.431	133.0131	133.0137	−4.5	100.0	C_4_H_6_O_5_	115.0013	Malic acid
2	0.476	191.0191	191.0192	−0.5	100.0	C_6_H_8_O_7_	111.0054	Citric acid
3	0.753	169.0138	169.0137	0.6	95.8	C_7_H_6_O_5_	125.0222	Gallic acid
4	1.247	153.0189	153.0188	0.7	100.0	C_7_H_6_O_4_	108.0185	Protocatehuic acid isomer a
5	1.688	175.0599	175.0606	−4.0	98.8	C_7_H_12_O_5_	113.0626	3-Isopropylmalic acid
6	3.32	289.0698	289.0712	−4.8	96.4	C_15_H_14_O_6_	179.0345	Catechin
7	3.500	153.0181	153.0188	−4.6	100.0	C_7_H_6_O_4_	-	Protocatehuic acid isomer b
8	4.009	705.1675	705.1667	1.1	95.8	C_32_H_34_O_18_	351.0702; 191.0544; 133.0271	Kaempferol 3-[2′′′,3′′′,5′′′-triacetyl-alpha-L-arabinofuranosyl-(1->6)-glucoside isomer a
9	4.009	353.0864	353.0873	−2.5	100.0	C_16_H_18_O_9_	191.0544	Chlorogenic acid isomer a
10	4.301	353.0869	353.0873	−1.1	100.0	C_16_H_18_O_9_	191.0543	Chlorogenic acid isomer b
11	4.368	705.1680	705.1667	1.8	92.1	C_32_H_34_O_18_	-	Kaempferol 3-[2′′′,3′′′,5′′′-triacetyl-alpha-L-arabinofuranosyl-(1->6)-glucoside isomer b
12	4.541	353.0859	353.0873	−4.0	97.5	C_16_H_18_O_9_	191.0538	Chlorogenic acid isomer c
13	4.69	289.0705	289.0712	−2.4	100.0	C_15_H_14_O_6_	245.0794	Epicatechin
14	4.952	337.0909	337.0923	−4.1	100.0	C_16_H_18_O_8_	173.0441	Coumaroylquinic acid isomer a
15	5.155	337.0914	337.0923	−2.7	100.0	C_16_H_18_O_8_	173.0439	Coumaroylquinic acid isomer a
16	5.678	579.1725	579.1714	1.9	93.1	C_27_H_32_O_14_	245.092	Narirutin
17	6.465	337.0546	337.0560	−4.2	99.9	C_15_H_14_O_9_	173.0082	Quercetin dihydrate
18	8.449	371.1332	371.1342	−2.7	99.6	C_17_H_24_O_9_	209.0801	Syringin isomer a
19	8.553	579.1719	579.1714	0.9	99.6	C_27_H_32_O_14_	271.0604; 167.0341	Naringin
20	8.606	463.0861	463.0877	−3.5	99.7	C_21_H_20_O_12_	300.0253; 271.0228; 167.0347	Isoquercetin
21	8.636	567.1716	567.1714	0.4	100.0	C_26_H_32_O_14_	463.0866; 300.0244;273.0748; 167.0342	Phloretin 2′-xyloglucoside
22	8.816	463.0872	463.0877	−1.1	100.0	C_21_H_20_O_12_	300.0256; 271.0240; 255.0276	Hyperoside
23	8.965	371.1340	371.1342	−0.5	100.0	C_17_H_24_O_9_	209.0811	Syringin isomer b
24	9.137	435.1301	435.1291	2.3	99.3	C_21_H_24_O_10_	273.0757	Phloridzin
25	9.137	609.1838	609.1819	3.1	96.9	C_28_H_34_O_15_	301.0711	Hesperidin
26	9.340	433.0771	433.0771	0.0	100.0	C_20_H_18_O_11_	300.0262; 271.0242; 241.0136	Quercetin 3-O-beta-D-xylopyranoside
27	9.827	447.0917	447.0927	−2.2	90.1	C_21_H_20_O_11_	285.0382; 255.0272; 227.0331	Kaempferol-3-glucoside
28	10.032	447.0932	447.0927	1.1	100.0	C_21_H_20_O_11_	300.0255; 271.0233	Quercetin 3-rhamnoside
29	10.111	461.0716	461.0720	−0.9	100.0	C_21_H_18_O_12_	285.0392	Kaempferol-3-glucuronide
30	10.942	489.1023	489.1033	−2.0	99.8	C_23_H_22_O_12_	285.0374; 255.0263; 227.0333	Kaempferol 3-(6-acetylgalactoside) isomer a
31	11.113	593.1883	593.1870	2.2	98.5	C_28_H_34_O_14_	285.076	Didymin
32	11.226	693.2756	693.2758	−0.3	99.1	C_34_H_46_O_15_	-	Nomilin glucoside
33	11.511	489.1056	489.1033	4.7	92.8	C_23_H_22_O_12_	285.0414; 255.0290; 227.0320	Kaempferol 3-(6-acetylgalactoside) isomer b
34	11.877	711.2866	711.2864	0.3	100.0	C_34_H_48_O_16_	607.276	Nomilinic acid 17-beta-D-glucopyranoside
35	12.139	271.0602	271.0606	−1.5	100.0	C_15_H_12_O_5_	151.001	Naringenin
36	12.640	345.0599	345.0610	−3.2	99.9	C_17_H_14_O_8_	287.0171	Limocitrin
37	12.648	301.0699	301.0712	−4.3	100.0	C_16_H_14_O_6_	164.0084	Hesperetin
38	13.240	385.1494	385.1499	−1.3	100.0	C_18_H_26_O_9_	223.096	Methylsyringin
39	13.712	529.2075	529.2074	0.2	100.0	C_28_H_34_O_10_	469.186	7-Acetoxy-6-hydroxylimonin
40	14.206	329.2328	329.2328	0.0	100.0	C_18_H_34_O_5_	211.1343	Pinellic acid

**Table 3:** Quantified phenolic compounds in the analyzed smoothies by HPLC-ESI-TOF-MS in µg/mL expressed as average ± standard deviation.

Phenolic Compound	S1	S2	S3	S4	S5	S6	S7	S8	S9	S10	S11	S12	S13	S14	S15	S16	S17	S18	S19	S20	S21	S22	S23
Gallic acid	367.25 ± 0.53	<LOQ	201.97 ± 0.36	5.56 ± 0.16	42.50 ± 0.08	32.47 ± 0.06	570.66 ± 0.74	62.27 ± 0.22	226.90 ± 0.39	964.22 ± 1.15	157.69 ± 0.32	405.54 ± 0.57	34.27 ± 0.07	41.18 ± 0.08	679.61 ± 0.85	<LOQ	31.44 ± 0.06	207.83 ± 0.37	40.15 ± 0.20	17.98 ± 0.04	543.29 ± 0.71	19.69 ± 0.05	<LOQ
Chlorogenic acid isomer a	532.92 ± 1.21	791.93 ± 1.48	1163.81 ± 1.86	673.74 ± 1.35	<LOQ	<LOQ	170.39 ± 0.84	230.75 ± 0.90	181.16 ± 0.85	500.54 ± 1.18	94.49 ± 0.76	160.96±0.51	697.92 ± 1.38	168.30 ± 0.53	1117.11 ± 1.81	859.87 ± 1.54	1226.82 ± 1.92	732.40 ± 1.41	713.90 ± 1.40	<LOQ	116.85 ± 0.79	185.17 ± 0.58	1144.12 ± 1.84
Chlorogenic acid isomer b	1232.31 ± 1.93	926.29 ± 1.61	784.19 ± 1.47	1015.41 ± 1.70	<LOQ	<LOQ	741.19 ± 1.42	581.39 ± 1.26	448.95 ± 1.12	999.52 ± 1.69	615.80 ± 1.30	354.76±1.03	1194.18 ± 1.89	318.21 ± 0.99	1090.78 ± 1.78	1052.55 ± 1.74	1353.11 ± 2.05	1189.69 ± 1.88	946.19 ± 1.63	<LOQ	409.35 ± 1.08	112.41 ± 0.78	1363.95 ± 2.06
Chlorogenic acid isomer c	45.73 ± 0.71	111.33 ± 0.36	523.06 ± 1.20	139.81 ± 0.45	<LOQ	<LOQ	81.93 ± 0.27	97.39 ± 0.32	105.24 ± 0.34	167.15 ± 0.53	106.08 ± 0.35	79.30±0.27	155.04 ± 0.49	39.27 ± 0.14	577.79 ± 1.26	95.03 ± 0.76	316.56 ± 0.99	166.47 ± 0.53	185.91 ± 0.59	<LOQ	80.20 ± 0.27	57.34 ± 0.20	139.74 ± 0.81
Coumaroylquinic acid isomer a	133.26 ± 0.43	145.68 ± 0.47	186.47 ± 0.59	122.63 ± 0.40	<LOQ	<LOQ	80.11 ± 0.27	37.49 ± 0.14	21.63 ± 0.09	65.14 ± 0.22	34.90 ± 0.13	115.07±0.37	136.37 ± 0.44	129.03 ± 0.42	1006.93 ± 1.69	187.89 ± 0.59	501.91 ± 1.18	139.30 ± 0.45	98.80 ± 0.32	<LOQ	59.14 ± 0.20	36.70 ± 0.14	204.54 ± 0.64
Coumaroylquinic acid isomer a	147.81 ± 0.47	157.45 ± 0.50	182.93 ± 0.58	131.98 ± 0.42	<LOQ	<LOQ	93.75 ± 0.31	35.58 ± 0.13	27.66 ± 0.11	59.72 ± 0.21	36.15 ± 0.14	141.90±0.45	200.41 ± 0.63	137.21 ± 0.44	1023.10 ± 1.71	189.33 ± 0.60	728.71 ± 1.41	154.30 ± 0.49	105.86 ± 0.35	<LOQ	81.45 ± 0.27	46.78 ± 0.17	199.41 ± 0.63
Protocatehuic acid isomer a	16.50 ± 0.04	12.11 ± 0.03	8.04 ± 0.16	392.01 ± 0.56	40.53 ± 0.08	<LOQ	16.13 ± 0.04	13.99 ± 0.04	0.08 ± 0.02	53.97 ± 0.10	95.67 ± 0.25	28.36±0.06	75.46 ± 0.23	4.62 ± 0.02	66.21 ± 0.12	33.88 ± 0.07	32.68 ± 0.07	25.71 ± 0.05	13.95 ± 0.04	<LOQ	26.49 ± 0.06	83.11 ± 0.14	12.14 ± 0.03
Protocatehuic acid isomer b	<LOQ	<LOQ	71.94 ± 0.12	688.17 ± 0.86	55.03 ± 0.10	<LOQ	<LOQ	128.77 ± 0.29	<LOQ	<LOQ	35.90 ± 0.07	<LOQ	49.74 ± 0.21	<LOQ	<LOQ	<LOQ	4.29 ± 0.02	<LOQ	<LOQ	<LOQ	<LOQ	95.95 ± 0.25	<LOQ
Catechin	138.09 ± 0.24	98.49 ± 0.13	165.39 ± 0.32	155.18 ± 0.29	440.95 ± 1.10	<LOQ	94.42 ± 0.12	490.36 ± 1.24	82.07 ± 0.09	128.59 ± 0.22	236.22 ± 0.52	68.69±0.05	100.04 ± 0.14	60.64 ± 0.03	172.85 ± 0.34	<LOQ	227.13 ± 0.50	177.63 ± 0.36	146.01 ± 0.27	<LOQ	88.55 ± 0.10	338.05 ± 0.81	150.17 ± 0.28
Epicatechin	412.15 ± 1.02	458.72 ± 1.15	338.92 ± 0.81	485.87 ± 1.23	81.51 ± 0.08	<LOQ	280.50 ± 0.65	408.96 ± 1.01	258.08 ± 0.58	370.42 ± 0.90	199.85 ± 0.42	91.06±0.11	105.29 ± 0.15	80.31 ± 0.08	417.27 ± 1.03	54.91 ± 0.01	222.64 ± 0.48	403.77 ± 1.00	498.76 ± 1.26	<LOQ	211.96 ± 0.45	267.64 ± 0.61	591.24 ± 1.53
Phloridzin	340.25 ± 1.02	685.88 ± 1.72	596.35 ± 1.54	365.08 ± 1.07	33.94 ± 0.07	<LOQ	20.00 ± 0.38	8.71 ± 0.35	50.23 ± 0.44	211.09 ± 0.76	100.02 ± 0.54	142.84±0.62	287.98 ± 0.92	80.44 ± 0.50	777.90 ± 1.91	687.57 ± 1.72	601.08 ± 1.55	304.90 ± 0.95	602.91 ± 1.55	<LOQ	54.16 ± 0.45	67.70 ± 0.27	504.35 ± 1.35
Phloretin 2′-xyloglucoside	36.34 ± 0.41	124.84 ± 0.59	55.60 ± 0.45	80.80 ± 0.31	<LOQ	<LOQ	48.35 ± 0.21	14.03 ± 0.12	32.17 ± 0.07	57.51 ± 0.16	7.16 ± 0.10	72.05±0.28	66.72 ± 0.27	37.06 ± 0.18	395.60 ± 1.14	116.66 ± 0.57	239.00 ± 0.82	78.62 ± 0.30	82.84 ± 0.31	<LOQ	30.77 ± 0.16	11.02 ± 0.11	102.55 ± 0.54
Narirutin	60.51 ± 0.25	32.44 ± 0.07	4.00 ± 0.09	7.12 ± 0.10	<LOQ	<LOQ	23.88 ± 0.04	18.94 ± 0.13	17.19 ± 0.01	0.72 ± 0.08	19.48 ± 0.02	16.25±0.01	22.43 ± 0.03	16.34 ± 0.01	37.25 ± 0.18	20.69 ± 0.02	31.65 ± 0.06	32.92 ± 0.07	32.12 ± 0.07	<LOQ	19.01 ± 0.02	<LOQ	19.21 ± 0.13
Naringin	63.75 ± 0.26	15.35 ± 0.01	<LOQ	18.90 ± 0.13	56.77 ± 0.45	19.39 ± 0.02	84.15 ± 0.51	<LOQ	<LOQ	47.25 ± 0.43	37.41 ± 0.18	147.01±0.63	28.06 ± 0.39	41.11 ± 0.42	<LOQ	<LOQ	<LOQ	124.72 ± 0.59	<LOQ	<LOQ	28.74 ± 0.39	15.45 ± 0.12	<LOQ
Hesperidin	206.93 ± 0.75	30.43 ± 0.06	<LOQ	53.01 ± 0.23	314.24 ± 0.97	28.60 ± 0.16	508.80 ± 1.36	<LOQ	<LOQ	332.79 ± 1.01	178.31 ± 0.70	554.81±1.46	305.55 ± 0.95	216.93 ± 0.77	<LOQ	27.08 ± 0.05	<LOQ	325.76 ± 0.99	<LOQ	31.24 ± 0.06	445.35 ± 1.24	77.77 ± 0.49	27.66 ± 0.05
Naringenin	<LOQ	19.28 ± 0.02	<LOQ	17.83 ± 0.01	16.77 ± 0.01	<LOQ	<LOQ	17.88 ± 0.01	<LOQ	<LOQ	21.46 ± 0.03	<LOQ	22.65 ± 0.03	22.41 ± 0.38	<LOQ	<LOQ	<LOQ	16.70 ± 0.01	<LOQ	<LOQ	17.15 ± 0.01	18.73 ± 0.02	<LOQ
Hesperetin	<LOQ	6.84 ± 0.10	<LOQ	<LOQ	16.78 ± 0.01	<LOQ	16.58 ± 0.01	<LOQ	<LOQ	<LOQ	15.57 ± 0.01	<LOQ	16.66 ± 0.01	492.79 ± 1.33	<LOQ	<LOQ	<LOQ	<LOQ	<LOQ	<LOQ	22.33 ± 0.03	16.34 ± 0.01	<LOQ
Didymin	57.14 ± 0.24	16.15 ± 0.01	<LOQ	1.41 ± 0.08	77.46 ± 0.30	18.08 ± 0.02	9.57 ± 0.36	<LOQ	<LOQ	28.22 ± 0.39	38.72 ± 0.19	26.27 ± 0.39	79.45 ± 0.30	80.07 ± 0.30	<LOQ	<LOQ	<LOQ	36.92 ± 0.41	<LOQ	<LOQ	2.50 ± 0.34	23.30 ± 0.14	<LOQ
Quercetin 3-rhamnoside	44.12 ± 0.43	234.36 ± 0.81	254.54 ± 0.85	122.60 ± 0.58	<LOQ	<LOQ	28.29 ± 0.16	11.14 ± 0.36	55.57 ± 0.23	25.54 ± 0.39	51.11 ± 0.22	26.86 ± 0.15	163.30 ± 0.67	67.58 ± 0.27	32.10 ± 0.40	84.19 ± 0.51	64.06 ± 0.26	74.14 ± 0.29	69.41 ± 0.48	<LOQ	26.95 ± 0.15	6.24 ± 0.09	59.61 ± 0.46
Quercetin dihydrate isomer a	18.52 ± 0.02	132.41 ± 0.60	<LOQ	19.32 ± 0.02	18.99 ± 0.02	13.29 ± 0.36	20.32 ± 0.02	<LOQ	15.36 ± 0.12	21.32 ± 0.03	15.97 ± 0.01	18.47 ± 0.02	19.38 ± 0.02	83.39 ± 0.50	<LOQ	<LOQ	<LOQ	20.12 ± 0.02	20.87 ± 0.03	36.51 ± 0.18	20.21 ± 0.02	<LOQ	47.17 ± 0.21
Quercetin 3-O-beta-D-xylopyranoside	56.29 ± 0.24	128.42 ± 0.60	171.28 ± 0.68	27.76 ± 0.39	<LOQ	<LOQ	28.82 ± 0.05	47.82 ± 0.21	29.40 ± 0.06	72.35 ± 0.21	20.07 ± 0.13	1.02 ± 0.08	44.44 ± 0.43	38.18 ± 0.18	13.94 ± 0.36	32.19 ± 0.17	81.85 ± 0.31	30.54 ± 0.16	86.18 ± 0.32	<LOQ	4.79 ± 0.09	33.83 ± 0.07	55.70 ± 0.23
Isoquercetin	30.88 ± 0.16	43.91 ± 0.42	392.84 ± 1.13	230.14 ± 0.80	<LOQ	<LOQ	20.98 ± 0.03	24.86 ± 0.04	31.90 ± 0.06	23.73 ± 0.14	15.68 ± 0.12	25.09 ± 0.04	120.26 ± 0.58	32.66 ± 0.07	33.30 ± 0.17	20.04 ± 0.13	50.82 ± 0.22	32.54 ± 0.07	39.32 ± 0.19	<LOQ	22.94 ± 0.03	33.71 ± 0.07	29.85 ± 0.16
Hyperoside	9.83 ± 0.10	43.32 ± 0.20	45.17 ± 0.43	18.91 ± 0.37	19.52 ± 0.13	<LOQ	22.11 ± 0.03	29.26 ± 0.16	22.93 ± 0.03	10.81 ± 0.11	52.70 ± 0.23	26.81 ± 0.05	115.12 ± 0.57	29.55 ± 0.06	26.45 ± 0.15	6.29 ± 0.09	38.57 ± 0.19	29.79 ± 0.06	11.91 ± 0.11	<LOQ	25.01 ± 0.04	64.15 ± 0.26	41.04 ± 0.19
Kaempferol-3-glucoside	<LOQ	18.38 ± 0.02	18.52 ± 0.13	25.60 ± 0.04	18.44 ± 0.02	<LOQ	<LOQ	72.85 ± 0.48	<LOQ	15.46 ± 0.01	32.47 ± 0.17	<LOQ	58.98 ± 0.24	19.29 ± 0.02	17.60 ± 0.01	<LOQ	31.91 ± 0.17	<LOQ	16.91 ± 0.01	<LOQ	<LOQ	58.21 ± 0.45	13.67 ± 0.11
Kaempferol-3-glucuronide	<LOQ	<LOQ	<LOQ	<LOQ	39.13 ± 0.41	<LOQ	<LOQ	89.21 ± 0.52	<LOQ	<LOQ	14.27 ± 0.12	<LOQ	<LOQ	<LOQ	<LOQ	<LOQ	38.55 ± 0.19	<LOQ	<LOQ	<LOQ	<LOQ	76.44 ± 0.29	<LOQ
Kaempferol 3-(6-acetylgalactoside) isomer a	<LOQ	<LOQ	<LOQ	<LOQ	54.85 ± 0.23	<LOQ	<LOQ	85.64 ± 0.51	<LOQ	<LOQ	25.44 ± 0.04	<LOQ	17.47 ± 0.01	<LOQ	<LOQ	<LOQ	32.62 ± 0.07	<LOQ	<LOQ	<LOQ	<LOQ	43.47 ± 0.20	17.21 ± 0.01
Kaempferol 3-(6-acetylgalactoside) isomer b	<LOQ	<LOQ	15.62 ± 0.01	<LOQ	28.65 ± 0.05	<LOQ	<LOQ	9.85 ± 0.10	<LOQ	<LOQ	19.49 ± 0.02	<LOQ	16.13 ± 0.01	18.00 ± 0.02	<LOQ	<LOQ	18.28 ± 0.02	<LOQ	<LOQ	<LOQ	<LOQ	24.13 ± 0.04	<LOQ
Kaempferol 3-[2′′′,3′′′,5′′′-triacetyl-alpha-L-arabinofuranosyl-(1->6)-glucoside isomer a	105.09 ± 0.55	177.64 ± 0.69	853.59 ± 2.06	173.70 ± 0.69	<LOQ	<LOQ	44.77 ± 0.20	25.33 ± 0.15	12.54 ± 0.11	69.02 ± 0.48	31.35 ± 0.16	25.93 ± 0.04	172.97 ± 0.69	1.81 ± 0.08	885.25 ± 2.12	162.14 ± 0.66	608.30 ± 1.56	124.20 ± 0.59	68.78 ± 0.47	<LOQ	11.83 ± 0.11	23.04 ± 0.03	312.60 ± 0.97
Kaempferol 3-[2′′′,3′′′,5′′′-triacetyl-alpha-L-arabinofuranosyl-(1->6)-glucoside isomer b	466.33 ± 1.28	363.52 ± 1.07	1278.43 ± 2.92	372.00 ± 1.09	<LOQ	<LOQ	48.46 ± 0.43	4.13 ± 0.34	68.34 ± 0.27	298.90 ± 0.94	47.80 ± 0.43	30.47 ± 0.16	525.69 ± 1.40	24.02 ± 0.14	1390.73 ± 3.15	467.01 ± 1.28	1161.21 ± 2.68	360.48 ± 1.06	220.77 ± 0.78	<LOQ	61.33 ± 0.25	3.55 ± 0.09	699.38 ± 1.75
Sum of phenolic acids	2475.79 ± 5.32	2144.80 ± 4.45	3122.40 ± 6.34	3169.31 ± 5.91	138.05 ± 0.26	32.47 ± 0.06	1754.16 ± 3.90	1187.63 ± 3.30	1011.62 ± 2.93	2810.25 ± 5.07	1176.67 ± 3.31	1285.88 ± 3.26	2543.38 ± 5.33	837.81 ± 2.63	5561.52 ± 9.22	2418.55 ± 5.31	4195.52 ± 7.70	2615.70 ± 5.19	2104.76 ± 4.52	17.98 ± 0.04	1316.76 ± 3.38	637.14 ± 2.31	3063.90 ± 6.01
Sum of flavonoids	2046.21 ± 6.96	2630.38 ± 8.26	4190.25 ± 11.41	2175.23 ± 7.43	1217.99 ± 3.86	79.36 ± 0.56	1300.01 ± 4.56	1358.97 ± 5.74	675.77 ± 2.07	1713.71 ± 6.24	1180.55 ± 4.35	1273.63 ± 4.09	2288.59 ± 7.80	1442.58 ± 5.35	4200.24 ± 10.97	1819.87 ± 5.22	3447.67 ± 9.07	2173.75 ± 6.92	1896.80 ± 5.85	67.75 ± 0.24	1093.58 ± 3.90	1202.77 ± 4.17	2671.39 ± 7.98
Sum of phenolic compounds	4521.99 ± 12.29	4775.17 ± 12.71	7312.65 ± 17.75	5344.54 ± 13.34	1356.04 ± 4.12	111.82 ± 0.62	3054.18 ± 8.45	2546.60 ± 9.03	1687.39 ± 5.00	4523.96 ± 11.31	2357.22 ± 7.66	2559.51 ± 7.36	4831.97 ± 13.13	2280.39 ± 7.97	9761.76 ± 20.19	4238.42 ± 10.53	7643.19 ± 16.76	4789.45 ± 12.11	4001.56 ± 10.37	85.73 ± 0.29	2410.34 ± 7.28	1839.90 ± 6.48	5735.29 ± 13.99

LOD: limit of detection; LOQ: limit of quantification.

**Table 4:** Vitamin C content of the smoothies analyzed by HPLC-UV/VIS expressed as average ± standard deviation.

Smoothie	Ascorbic Acid	Dehidroascorbic Acid	Total Vitamin C
	µg AA/mL Smoothie	µg AA/mL Smoothie	µg AA/mL Smoothie
1	797.00 ± 2.93	<LOD	797.00 ± 2.93
2	619.77 ± 2.52	80.21 ± 0.20	699.98 ± 2.72
3	383.53 ± 1.97	164.98 ± 0.80	548.51 ± 2.77
4	293.28 ± 1.76	254.43 ± 1.44	547.71 ± 3.20
5	562.09 ± 2.38	<LOD	562.09 ± 2.38
6	556.29 ± 2.37	<LOD	556.29 ± 2.37
7	556.59 ± 2.37	96.03 ± 0.04	652.61 ± 2.41
8	328.37 ± 1.84	195.52 ± 1.08	523.89 ± 2.93
9	2397.80 ± 6.63	262.22 ± 4.09	2660.02 ± 10.73
10	923.67 ± 3.22	48.75 ± 1.14	972.42 ± 4.36
11	390.51 ± 1.99	133.37 ± 0.64	523.89 ± 2.63
12	651.14 ± 2.59	29.30 ± 0.51	680.45 ± 3.10
13	500.68 ± 2.24	77.00 ± 0.10	577.68 ± 2.34
14	947.16 ± 3.28	11.34 ± 1.37	958.50 ± 4.65
15	560.45 ± 2.38	102.06 ± 0.05	662.52 ± 2.44
16	<LOD	491.78 ± 3.26	491.78 ± 3.26
17	542.91 ± 2.34	84.01 ± 0.02	626.92 ± 2.36
18	1196.64 ± 3.85	<LOD	1196.64 ± 3.85
19	432.14 ± 2.08	59.63 ± 0.21	491.78 ± 2.29
20	518.97 ± 2.29	40.24 ± 0.11	559.21 ± 2.39
21	419.06 ± 2.05	144.70 ± 0.62	563.76 ± 2.67
22	489.38 ± 2.22	51.36 ± 0.02	540.75 ± 2.23
23	639.85 ± 2.56	27.22 ± 0.49	667.07 ± 3.05

AA: ascorbic acid.

**Table 5:** Antioxidant activities of the smoothies expressed as average ± standard deviation.

Smoothie	DPPH	FRAP
	µg TE/mL Smoothie	µg TE/mL Smoothie
1	865.25 ± 2.19	4322.37 ± 17.83
2	562.50 ± 9.19	3041.58 ± 11.83
3	1926.50 ± 4.38	6779.47 ± 13.47
4	1633.75 ± 11.53	5448.16 ± 71.94
5	1085.50 ± 5.37	3942.63 ± 26.13
6	495.00 ± 5.66	2648.95 ± 12.65
7	676.50 ± 30.26	3557.37 ± 0.67
8	1591.50 ± 0.99	7271.05 ± 20.77
9	1314.00 ± 20.08	8167.37 ± 78.60
10	1105.00 ± 5.23	4828.68 ± 35.84
11	1685.50 ± 17.82	6551.84 ± 37.25
12	580.00 ± 8.77	3033.68 ± 10.64
13	1847.25 ± 5.73	5945.79 ± 61.93
14	762.75 ± 0.64	2873.68 ± 9.30
15	1058.50 ± 8.06	4718.68 ± 11.05
16	220.50 ± 5.23	1126.84 ± 7.29
17	1477.50 ± 13.72	5527.89 ± 75.85
18	1012.75 ± 6.72	4656.84 ± 11.61
19	663.75 ± 6.01	2736.05 ± 5.99
20	638.75 ± 10.96	3247.37 ± 1.27
21	597.25 ± 6.29	3286.32 ± 22.70
22	1637.00 ± 16.12	5915.26 ± 6.48
23	603.40 ± 11.48	3261.84 ± 30.85

TE: Trolox equivalents.

**Table 6:** List of the smoothies analyzed with their code name and their ingredients.

Code	Ingredients of the Smoothie
1	Apple, orange, goji, passion fruit, mango, banana
2	Pineapple, lime, apple, mint, chlorophyll
3	Apple, blueberry, banana, pomegranate, grape, currant
4	Banana, grape, apple, cranberry, orange, pomegranate, acai, chokeberry, lemon
5	Strawberry, banana, grape, orange
6	Pineapple, banana, coconut, mango, lemon
7	Apple, banana, mango, orange, passion fruit
8	Strawberry, apple, banana
9	Apple, pineapple, mango, carrot, coconut
10	Apple, mango, banana, orange, passion fruit, peach, lemon
11	Apple, banana, grape, strawberry, blackberry, raspberry, orange, currant
12	Orange, apple, banana, mango, passion fruit
13	Apple, banana, orange, raspberry, blueberry, blackberry, blackcurrant, redcurrant
14	Pineapple, apple, orange, banana, lime, spirulina, mint
15	Apple, mango, banana, passion fruit
16	Apple, cucumber, celery, kale, spinach, lemon, ginger
17	Apple, strawberry, raspberry, blueberry, banana
18	Apple, mango, banana, orange, passion fruit, peach, lime
19	Apple, pineapple, mango, coconut, banana
20	Pineapple, banana, coconut, mango, lemon
21	Apple, mango, coconut, banana, orange, passion fruit
22	Strawberry, apple, banana, coconut, blackberry, blackcurrant, orange
23	Apple, pineapple, pear, kiwi, lime, spirulina

## Data Availability

Not applicable.

## Supplementary Materials

The following supporting information can be downloaded at: https://www.mdpi.com/article/10.3390/molecules27238229/s1, Figure S1: Total ion chromatogram of analysed smoothies S3, S8, S9, S14, S15 and S18 by HPLC-ESI-MS-TOF. Figure S2: Pearson’s correlation heatmap between smoothie’s ingredients, nutritional features, and antioxidant and phenol content. Table S1: Standard analytes used for elaborating the calibration curves with the equations, R^2^, LOD, and LOQ of each compound.

## References

[1] Trichopoulou A., Lagiou P. (1997). Healthy traditional Mediterranean diet: An expression of culture, history, and lifestyle. Nutr. Rev..

[2] Dinu M., Pagliai G., Casini A., Sofi F. (2018). Mediterranean diet and multiple health outcomes: An umbrella review of meta-analyses of observational studies and randomised trials. Eur. J. Clin. Nutr..

[3] Carlos S., De La Fuente-Arrillaga C., Bes-Rastrollo M., Razquin C., Rico-Campà A., Martínez-González M.A., Ruiz-Canela M. (2018). Mediterranean diet and health outcomes in the SUN cohort. Nutrients.

[4] Soltani S., Arablou T., Jayedi A., Salehi-Abargouei A. (2020). Adherence to the dietary approaches to stop hypertension (DASH) diet in relation to all-cause and cause-specific mortality: A systematic review and dose-response meta-analysis of prospective cohort studies. Nutr. J..

[5] Pressler M., Devinsky J., Duster M., Lee J.H., Glick C.S., Wiener S., Laze J., Friedman D., Roberts T., Devinsky O. (2022). Dietary Transitions and Health Outcomes in Four Populations—Systematic Review. Front. Nutr..

[6] Kyriacou A., Evans J.M.M., Economides N., Kyriacou A. (2015). Adherence to the Mediterranean diet by the Greek and Cypriot population: A systematic review. Eur. J. Public Health.

[7] Obeid C.A., Gubbels J.S., Jaalouk D., Kremers S.P.J., Oenema A. (2022). Adherence to the Mediterranean diet among adults in Mediterranean countries: A systematic literature review. Eur. J. Nutr..

[8] Nyberg S.T., Batty G.D., Pentti J., Virtanen M., Alfredsson L., Fransson E.I., Goldberg M., Heikkilä K., Jokela M., Knutsson A. (2018). Obesity and loss of disease-free years owing to major non-communicable diseases: A multicohort study. Lancet Public Health.

[9] Afshin A., Sur P.J., Fay K.A., Cornaby L., Ferrara G., Salama J.S., Mullany E.C., Abate K.H., Abbafati C., Abebe Z. (2019). Health effects of dietary risks in 195 countries, 1990–2017: A systematic analysis for the Global Burden of Disease Study 2017. Lancet.

[10] Janssen F., Trias-Llimós S., Kunst A.E. (2021). The combined impact of smoking, obesity and alcohol on life-expectancy trends in Europe. Int. J. Epidemiol..

[11] Hirashiki A., Shimizu A., Nomoto K., Kokubo M., Suzuki N., Arai H. (2022). Systematic Review of the Effectiveness of Community Intervention and Health Promotion Programs for the Prevention of Non-Communicable Diseases in Japan and Other East and Southeast Asian Countries. Circ. Rep..

[12] Septembre-Malaterre A., Remize F., Poucheret P. (2018). Fruits and vegetables, as a source of nutritional compounds and phytochemicals: Changes in bioactive compounds during lactic fermentation. Food Res. Int..

[13] Román G.C., Jackson R.E., Gadhia R., Román A.N., Reis J. (2019). Mediterranean diet: The role of long-chain ω-3 fatty acids in fish; polyphenols in fruits, vegetables, cereals, coffee, tea, cacao and wine; probiotics and vitamins in prevention of stroke, age-related cognitive decline, and Alzheimer disease. Rev. Neurol..

[14] Angiolillo L., Del Nobile M.A., Conte A. (2015). The extraction of bioactive compounds from food residues using microwaves. Curr. Opin. Food Sci..

[15] Tiwari U., Cummins E. (2013). Factors influencing levels of phytochemicals in selected fruit and vegetables during pre- and post-harvest food processing operations. Food Res. Int..

[16] Liu R.H. (2013). Dietary bioactive compounds and their health implications. J. Food Sci..

[17] Liu R.H. (2013). Health-promoting components of fruits and vegetables in the diet. Adv. Nutr..

[18] Martínez-Navarrete N., del Mar Camacho Vidal M., José Martínez Lahuerta J. (2008). Los compuestos bioactivos de las frutas y sus efectos en la salud. Act. Diet..

[19] Pattern H.P.D., Bo C.D., Bernardi S., Marino M., Porrini M., Tucci M., Guglielmetti S., Cherubini A., Carrieri B., Kirkup B. (2019). Systematic Review on Polyphenol Intake and Health Outcomes: Is there Sufficient Evidence to Define a Health-Promoting Polyphenol-Rich Dietary Pattern?. Nutrients.

[20] Ngo B., Van Riper J.M., Cantley L.C., Yun J. (2019). Targeting cancer vulnerabilities with high-dose vitamin C. Nat. Rev. Cancer.

[21] Gordon D.S., Rudinsky A.J., Guillaumin J., Parker V.J., Creighton K.J. (2020). Vitamin C in Health and Disease: A Companion Animal Focus. Top. Companion Anim. Med..

[22] Doseděl M., Jirkovský E., Macáková K., Krčmová L.K., Javorská L., Pourová J., Mercolini L., Remião F., Nováková L., Mladěnka P. (2021). Vitamin c—Sources, physiological role, kinetics, deficiency, use, toxicity, and determination. Nutrients.

[23] Padayatty S.J., Levine M. (2016). Vitamin C: The known and the unknown and Goldilocks. Oral Dis..

[24] Dowling D.D., Duerbeck J.M. (2016). Vitamin C: Promises Not Kept. Obstet. Gynecol. Surv..

[25] WHO (2020). Healthy Diet.

[26] Van de Velde F., Vignatti C., Paula Méndez-Galarraga M., Gomila M., Fenoglio C., Donda Zbinden M., Élida Pirovani M. (2022). Intestinal and colonic bioaccessibility of phenolic compounds from fruit smoothies as affected by the thermal processing and the storage conditions. Food Res. Int..

[27] Di Cagno R., Minervini G., Rizzello C.G., De Angelis M., Gobbetti M. (2011). Effect of lactic acid fermentation on antioxidant, texture, color and sensory properties of red and green smoothies. Food Microbiol..

[28] Artés-Hernández F., Martínez-Hernández G.B., Aguayo E., Gómez P.A., Castillejo N., Arjmandi M., Formica-Oliveira C., González-Tejedor G., Otón M., Pedreño J.L. (2016). Smoothies: Nueva moda saludable de consumo de productos hortofrutícolas con elevado valor nutritivo. Retos tecnológicos para la industria. CTC Aliment. Rev. Sobre Agroaliment. Ind. Afines.

[29] Cano-Lamadrid M., Tkacz K., Turkiewicz I.P., Clemente-Villalba J., Sánchez-Rodríguez L., Lipan L., García-García E., Carbonell-Barrachina Á.A., Wojdyło A. (2020). How a Spanish group of millennial generation perceives the commercial novel smoothies?. Foods.

[30] Castillejo N., Martínez-Hernández G.B., Gómez P.A., Artés F., Artés-Hernández F. (2016). Red fresh vegetables smoothies with extended shelf life as an innovative source of health-promoting compounds. J. Food Sci. Technol..

[31] Rodríguez-Verástegui L.L., Martínez-Hernández G.B., Castillejo N., Gómez P.A., Artés F., Artés-Hernández F. (2015). Bioactive Compounds and Enzymatic Activity of Red Vegetable Smoothies During Storage. Food Bioprocess Technol..

[32] Petruzzi L., Campaniello D., Speranza B., Corbo M.R., Sinigaglia M., Bevilacqua A. (2017). Thermal Treatments for Fruit and Vegetable Juices and Beverages: A Literature Overview. Compr. Rev. Food Sci. Food Saf..

[33] Ferreira J.A., Santos J.M., Breitkreitz M.C., Ferreira J.M.S., Lins P.M.P., Farias S.C., de Morais D.R., Eberlin M.N., Bottoli C.B.G. (2019). Characterization of the lipid profile from coconut (Cocos nucifera L.) oil of different varieties by electrospray ionization mass spectrometry associated with principal component analysis and independent component analysis. Food Res. Int..

[34] Tahmassebi J.F., Kandiah P., Sukeri S. (2014). The effects of fruit smoothies on enamel erosion. Eur. Arch. Paediatr. Dent..

[35] Li R., Wang Y., Wang S., Liao X. (2015). A Comparative Study of Changes in Microbiological Quality and Physicochemical Properties of N2-Infused and N2-Degassed Banana Smoothies After High Pressure Processing. Food Bioprocess Technol..

[36] Cano-Lamadrid M., Nowicka P., Hernández F., Carbonell-Barrachina A., Wojdyło A. (2019). Quality of new healthy smoothies based on pomegranate and minor Mediterranean fruits. Acta Hortic..

[37] de Moura S.C.S.R., Vissotto F.Z., Berbari S.A.G., Souza E.D.C.G., Toti F.G.P., Alves Júnior P. (2017). Characterization and evaluation of stability of bioactive compounds in fruit smoothies. Food Sci. Technol..

[38] Tkacz K., Wojdyło A., Turkiewicz I.P., Nowicka P. (2021). Anti-diabetic, anti-cholinesterase, and antioxidant potential, chemical composition and sensory evaluation of novel sea buckthorn-based smoothies. Food Chem..

[39] Nowicka P., Wojdyło A., Teleszko M. (2017). Effect of mixing different kinds of fruit juice with sour cherry puree on nutritional properties. J. Food Sci. Technol..

[40] Li M., Zhang L., Zhang Q., Zi X., Lv R., Tang J., Zhou H. (2020). Impacts of Citric Acid and Malic Acid on Fermentation Quality and Bacterial Community of Cassava Foliage Silage. Front. Microbiol..

[41] Ricciutelli M., Moretti S., Galarini R., Sagratini G., Mari M., Lucarini S., Vittori S., Caprioli G. (2019). Identification and quantification of new isomers of isopropyl-malic acid in wine by LC-IT and LC-Q-Orbitrap. Food Chem..

[42] Aruwa C.E., Amoo S., Kudanga T. (2019). Phenolic compound profile and biological activities of Southern African Opuntia ficus-indica fruit pulp and peels. LWT.

[43] Rothwell J.A., Perez-Jimenez J., Neveu V., Medina-Remón A., M’hiri N., García-Lobato P., Manach C., Knox C., Eisner R., Wishart D.S. (2013). Phenol-Explorer 3.0: A major update of the Phenol-Explorer database to incorporate data on the effects of food processing on polyphenol content. Database.

[44] Schieber A., Keller P., Carle R. (2001). Determination of phenolic acids and flavonoids of apple and pear by high-performance liquid chromatography. J. Chromatogr. A.

[45] Al-Farsi M., Alasalvar C., Morris A., Baron M., Shahidi F. (2005). Comparison of antioxidant activity, anthocyanins, carotenoids, and phenolics of three native fresh and sun-dried date (*Phoenix dactylifera* L.) varieties grown in Oman. J. Agric. Food Chem..

[46] Meinhart A.D., Damin F.M., Caldeirão L., de Jesus Filho M., da Silva L.C., da Silva Constant L., Filho J.T., Wagner R., Godoy H.T. (2019). Chlorogenic and caffeic acids in 64 fruits consumed in Brazil. Food Chem..

[47] Neveu V., Perez-Jiménez J., Vos F., Crespy V., du Chaffaut L., Mennen L., Knox C., Eisner R., Cruz J., Wishart D. (2010). Phenol-Explorer: An online comprehensive database on polyphenol contents in foods. Database.

[48] Egüés I., Hernandez-Ramos F., Rivilla I., Labidi J. (2021). Optimization of ultrasound assisted extraction of bioactive compounds from apple pomace. Molecules.

[49] Razola-Díaz M.D.C., Guerra-Hernández E.J., Rodríguez-Pérez C., Gómez-Caravaca A.M., García-Villanova B., Verardo V. (2021). Optimization of Ultrasound-Assisted Extraction via Sonotrode of Phenolic Compounds from Orange By-Products. Foods.

[50] Wang S.Y., Zheng W., Galletta G.J. (2002). Cultural system affects fruit quality and antioxidant capacity in strawberries. J. Agric. Food Chem..

[51] Rommel A., Wrolstad R.E. (1993). Composition of Flavonols in Red Raspberry Juice As Influenced by Cultivar, Processing, and Environmental Factors. J. Agric. Food Chem..

[52] Gliszczynska-Swiglo A., Tyrakowska B. (2003). Quality of commercial apple juices evaluated on the basis of the polyphenol content and the TEAC antioxidant activity. J. Food Sci..

[53] Gualdani R., Cavalluzzi M., Lentini G., Habtemariam S. (2016). The Chemistry and Pharmacology of Citrus Limonoids. Molecules.

[54] Shi Y.-S.S., Zhang Y., Li H.-T.T., Wu C.-H.H., El-Seedi H.R., Ye W.-K.K., Wang Z.-W.W., Li C.-B.B., Zhang X.-F.F., Kai G.-Y.Y. (2020). Limonoids from Citrus: Chemistry, anti-tumor potential, and other bioactivities. J. Funct. Foods.

[55] Müller L., Gnoyke S., Popken A.M., Böhm V. (2010). Antioxidant capacity and related parameters of different fruit formulations. LWT Food Sci. Technol..

[56] Teleszko M., Wojdylo A. (2014). Bioactive compounds vs. organoleptic assessment of ’smoothies’-type products prepared from selected fruit species. Int. J. Food Sci. Technol..

[57] Nowicka P., Wojdyło A., Teleszko M., Samoticha J., Th P., Wojdyło A., Teleszko M., Samoticha J. (2016). Sensory attributes and changes of physicochemical properties during storage of smoothies prepared from selected fruit. LWT.

[58] Villagrán M., Muñoz M., Díaz F., Troncoso C., Celis-Morales C., Mardones L. (2019). Vitamin c in health and disease: A current perspective. Rev. Chil. Nutr..

[59] Hurtado A., Picouet P., Jofré A., Guàrdia M.D., Ros J.M., Bañón S. (2015). Application of High Pressure Processing for Obtaining “Fresh-Like” Fruit Smoothies. Food Bioprocess Technol..

[60] Keenan D.F., Brunton N.P., Gormley T.R., Butler F., Tiwari B.K., Patras A. (2010). Effect of thermal and high hydrostatic pressure processing on antioxidant activity and colour of fruit smoothies. Innov. Food Sci. Emerg. Technol..

[61] González-Tejedor G.A., Martínez-Hernández G.B., Garre A., Egea J.A., Fernández P.S., Artés-Hernández F. (2017). Quality Changes and Shelf-Life Prediction of a Fresh Fruit and Vegetable Purple Smoothie. Food Bioprocess Technol..

[62] Andrés V., Villanueva M.J., Tenorio M.D. (2016). The effect of high-pressure processing on colour, bioactive compounds, and antioxidant activity in smoothies during refrigerated storage. Food Chem..

[63] Bestwick C., Scobbie L., Milne L., Duncan G., Cantlay L., Russell W. (2020). Fruit-Based beverages contain a wide range of phytochemicals and intervention targets should account for the individual compounds present and their availability. Foods.

[64] Hurtado A., Guàrdia M.D., Picouet P., Jofré A., Ros J.M., Bañón S. (2017). Stabilisation of red fruit-based smoothies by high-pressure processing. Part II: Effects on sensory quality and selected nutrients. J. Sci. Food Agric..

[65] Keenan D.F., Brunton N., Gormley R., Butler F. (2011). Effects of thermal and high hydrostatic pressure processing and storage on the content of polyphenols and some quality attributes of fruit smoothies. J. Agric. Food Chem..

[66] Škegro M., Putnik P., Bursać Kovačević D., Kovač A.P., Salkić L., Čanak I., Frece J., Zavadlav S., Ježek D. (2021). Chemometric Comparison of High-Pressure Processing and Thermal Pasteurization: The Nutritive, Sensory, and Microbial Quality of Smoothies. Foods.

[67] Verni M., Pontonio E., Krona A., Jacob S., Pinto D., Rinaldi F., Verardo V., Díaz-de-Cerio E., Coda R., Rizzello C.G. (2020). Bioprocessing of Brewers’ Spent Grain Enhances Its Antioxidant Activity: Characterization of Phenolic Compounds and Bioactive Peptides. Front. Microbiol..

[68] Mesías-García M., Guerra-Hernández E., García-Villanova B. (2010). Determination of furan precursors and some thermal damage markers in baby foods: Ascorbic acid, dehydroascorbic acid, hydroxymethylfurfural and furfural. J. Agric. Food Chem..

[69] Brand-Williams W., Cuvelier M.E., Berset C. (1995). Use of a free redical method to evaluate antioxidant activity. LWT Food Sci. Technol..

[70] Parejo I., Codina C., Petrakis C., Kefalas P. (2000). Evaluation of scavenging activity assessed by Co(II)/EDTA-induced luminol chemiluminescence and DPPH· (2,2-diphenyl-1-picrylhydrazyl) free radical assay. J. Pharmacol. Toxicol. Methods.

[71] Pulido R., Bravo L., Saura-Calixto F. (2000). Antioxidant activity of dietary polyphenols as determined by a modified ferric reducing/antioxidant power assay. J. Agric. Food Chem..

