# Supplementary material for: New Insight on Phenolic Composition and Evaluation of the Vitamin C and Nutritional Value of Smoothies Sold on the Spanish Market
Source: Molecules. 2022 Nov 25;27(23):8229. doi: 10.3390/molecules27238229 (PMC9739094; doi:10.3390/molecules27238229)
Supplement: Supplementary file 1 [file molecules-27-08229-s001.zip › molecules-2014077-supplementary.pdf]

molecules

MDPI

Supplementary Materials

New Insight on Phenolic Composition and Evaluation of the Vitamin C and Nutritional Value of Smoothies Sold on the Spanish Market

María del Carmen Razola-Díaz [1,2], Eduardo Jesús Guerra-Hernández [1], Belén García-Villanova [1] and Vito Verardo [1,2,*]

[1] Department of Nutrition and Food Science, Campus of Cartuja, University of Granada, 18011 Granada, Spain
[2] Institute of Nutrition and Food Technology "José Mataix", Biomedical Research Center, University of Granada, Avda del Conocimiento sn., Armilla, 18100 Granada, Spain
* Correspondence: vitoverardo@ugr.es

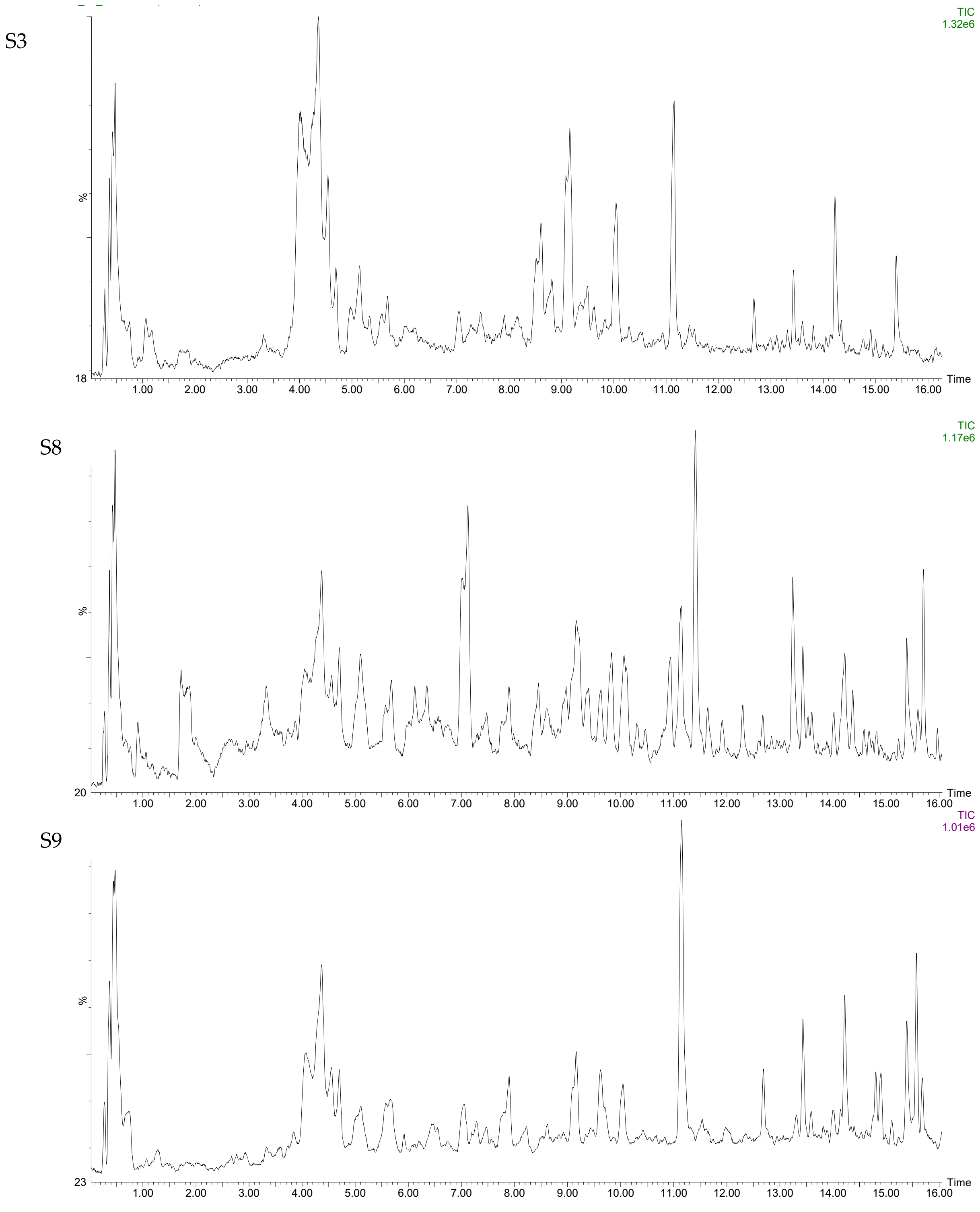

Figure S1. Total ion chromatogram of analysed smoothies S3, S8, S9, S14, S15 and S18 by HPLC-ESI-MS-TOF.

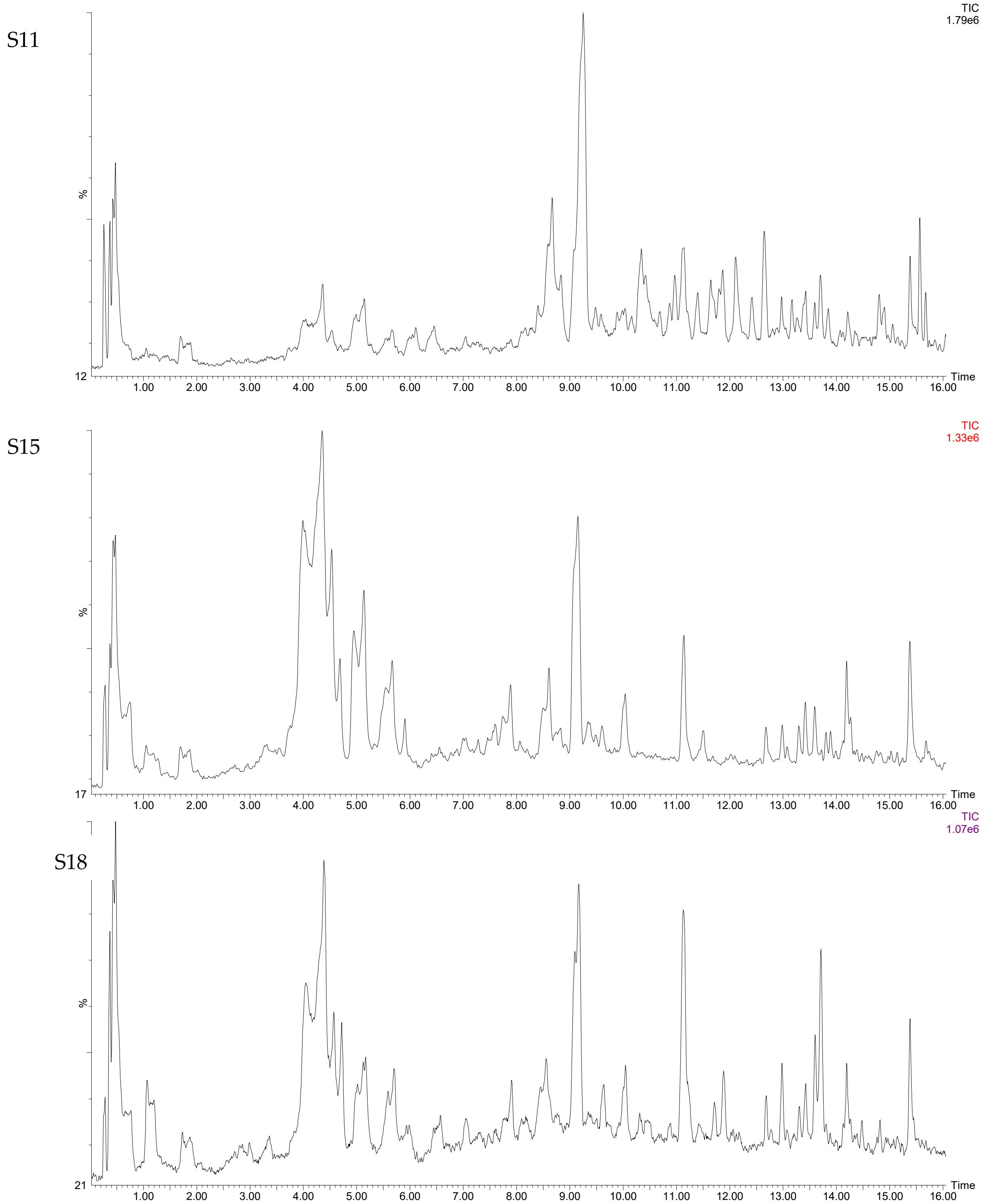

Figure S1. (continuation). Total ion chromatogram of analysed smoothies S3, S8, S9, S14, S15 and S18 by HPLC-ESI-MS-TOF.

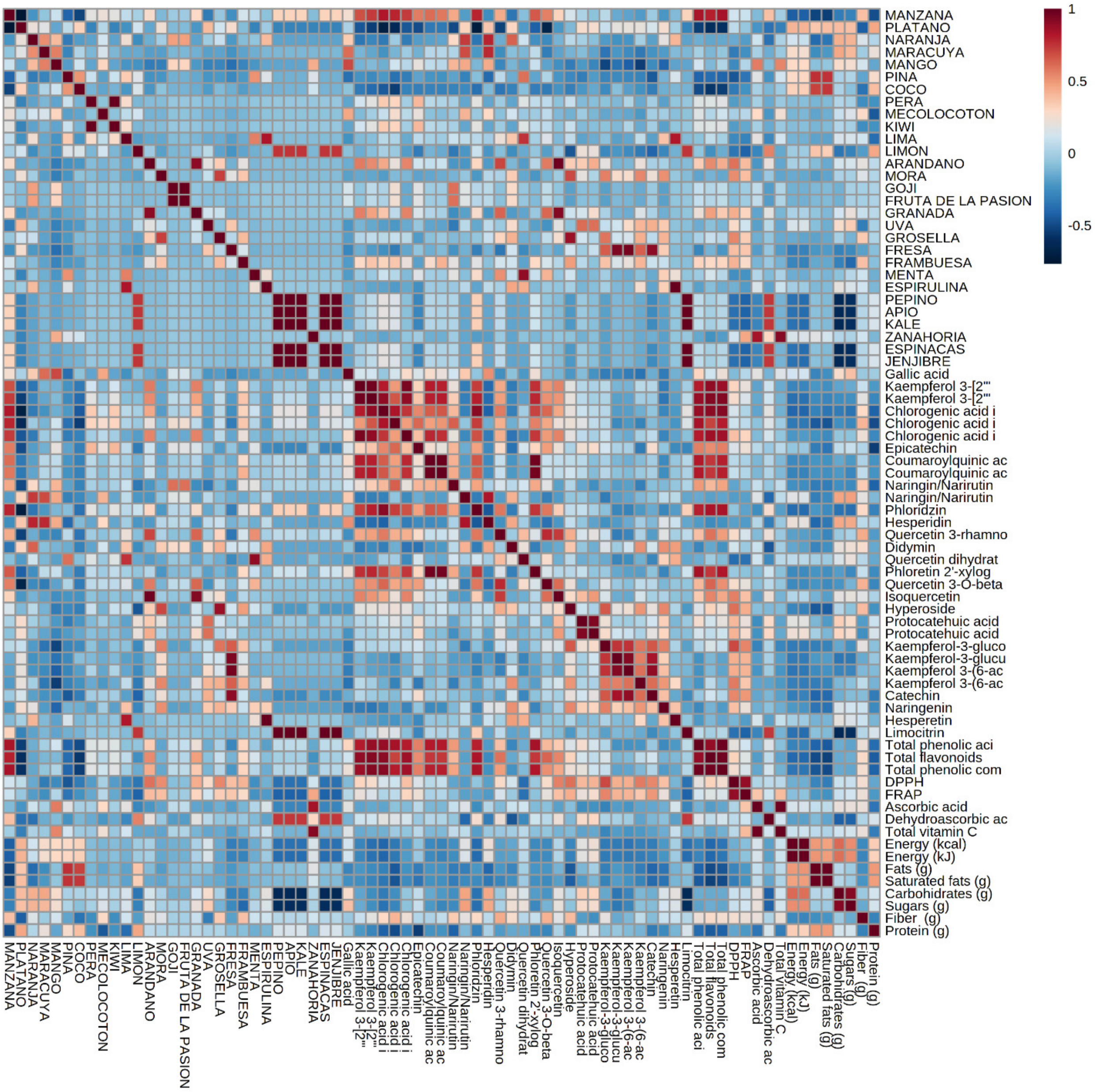

Figure S2. Pearson's correlation heatmap between smoothie's ingredients, nutritional features, and antioxidant and phenol content.

Table S1. Standard analytes used for elaborating the calibration curves with the equations, R^2, LOD, and LOQ of each compound.

Standard	LOD (μg/mL)	LOQ (μg/mL)	Calibration Curve (μg/mL)	R^2
Vanillic acid	7.57	25.25	y = 276.25x + 233.48	0.9874
Chlorogenic acid	3.84	12.79	y = 545.24x + 371.66	0.9984
Ferulic acid	6.69	22.29	y = 312.87x + 9852.2	0.9843
Quercetin	6.58	21.95	y = 317.77x + 5082.8	0.9982
Catechin	4.09	13.62	y = 512.18x - 2064.9	0.9959
Rutin	3.27	10.90	y = 639.83x - 685.16	0.9944
Phloretin	2.94	9.79	y = 712.07x + 970.95	0.9857
Phloridzin	6.79	22.65	y = 307.97x + 1009.8	0.9809
